# Prototype Effect and the Persuasiveness of Generalizations

**DOI:** 10.1007/s13164-015-0264-1

**Published:** 2015-05-06

**Authors:** Christian Dahlman, Farhan Sarwar, Rasmus Bååth, Lena Wahlberg, Sverker Sikström

**Affiliations:** Lund University, Lund, Sweden

## Abstract

An argument that makes use of a generalization activates the prototype for the category used in the generalization. We conducted two experiments that investigated how the activation of the prototype affects the persuasiveness of the argument. The results of the experiments suggest that the features of the prototype overshadow and partly overwrite the actual facts of the case. The case is, to some extent, judged as if it had the features of the prototype instead of the features it actually has. This prototype effect increases the persuasiveness of the argument in situations where the audience finds the judgment more warranted for the prototype than for the actual case (positive prototype effect), but decreases persuasiveness in situations where the audience finds the judgment less warranted for the prototype than for the actual case (negative prototype effect).

## Introduction

This study is concerned with arguments that make use of a generalization to make a judgment in a specific case. As an example, imagine a criminal trial where a 7-year-old girl testifies as a witness for the prosecution and the defense attorney questions her reliability with the argument that “a child is less reliable as a witness”. We are interested in the persuasiveness of such generalizations, and investigate how persuasiveness is related to *prototype effects*.

A generalization claims that cases belonging to a certain category (e.g. “child”) have a certain property (e.g. “less reliable as a witness”). According to *prototype theory* some of the cases that belong to a certain category are more typical members of that category than other cases (Rosch 1975; Lakoff 1987; Langacker 1987; Gärdenfors 2000). A 7-year-old is, for example, a more typical member of the category “child” than a 14-year-old. In prototype theory, the most typical member of a category is referred to as the *prototype* of the category in question. It should be pointed out that a prototype is an abstract entity, not an actual case. The “child” prototype is not an actual child, but an imaginary child with all the features that are typically associated with children (youth, playfulness, lack of responsibility etc). A prototype is a mental representation that serves as a cognitive reference point for the category. The most salient features of the prototype are the first features that come to mind when the category is mentioned. The effects that prototypes have on categorization are referred to as *prototype effects*. That the response time for categorization is shorter for cases that fit the prototype is an example of a prototype effect (Lakoff 1987).

Previous studies on prototypes show that subjects are more likely to apply a generalization to a case if the case fits the prototype for the category used in the generalization (Inman and Baron 1996; Inman et al. 1998; Krumm and Corning 2008). Our study is concerned with situations where a generalization is used in argumentation to make an audience agree with a certain judgment, and we investigate to what extent the agreement is influenced by prototype effects. Some cases fit a prototype better than other cases, and we examine to what extent such differences have an effect on the persuasiveness of the generalization.

## Positive and Negative Prototype Effects

An argument that makes use of a generalization claims that cases belonging to a certain category have a certain property (e.g. “a child is less reliable as a witness”), and applies this generalization to a specific case. If we look at cases where a certain generalization is applied, an audience will find the judgment more *warranted* in some cases than others (Toulmin 1958). When it comes to child witnesses, an audience will, for example, find the judgment that X is less reliable because X is a child, more warranted in a case where X is 6 years old than in a case where X is 8 years old (Ross et al. 1989, 1990). The warrantedness of a judgment can be assessed in a relation to the prototype of the category in question. Suppose, for example, that the “prototype child” is 7 years old. That would mean that the judgment that X is “less reliable as witness” is more warranted than the judgment that the “prototype child” is “less reliable as a witness” if X is under 7 years old, and less warranted if X is older than seven. The question is how this affects the persuasiveness of an argument that uses a generalization.

The activation of the prototype has the potential to overshadow the rest of the category, so that all members of the category are perceived to have the features of the prototype (Lakoff 1987, 85). This kind of prototype effect has been described in an article by Steven Winter as a “radical prototype effect” (Winter 1988, 1386), and in later works by Winter as an “assimilation-to-prototype-effect” (Winter 2001, 150–151). When the features of a prototype overshadow the actual facts of the case, the case will be judged as if it had the features of the prototype rather than the features it actually has. As an example, if the prototype child is 7 years old, an argument where the category “child” is applied to a 12-year-old would be judged as if the 12-year-old was a 7-year-old. This would make an audience *agree more* with the judgment, since the audience would find the argument that a 7-year-old is less reliable as a witness more warranted than the argument that a 12-year-old is less reliable. In the following, we will refer to such an effect as a *positive prototype effect*. In the opposite situation where the audience finds the judgment less warranted for the prototype than the actual case, a prototype that overshadows the actual facts of the case will make the audience *agree less* with the judgment. We will refer to this kind of effect as a *negative prototype effect*. The application of the category “child” to a 5-year-old could, for example, activate the prototypical 7-year-old child, to the effect that the 5-year-old is judged as if it was a 7-year-old. As a general theory, this connection between prototype effect and persuasiveness can be summarized as follows: If the audience finds the judgment *more warranted* for the prototype than for the actual case, the generalization will make the audience *agree more* with the judgment (positive prototype effect). If the audience finds the judgment *equally warranted* for the prototype and the actual case, the generalization will have *no effect* on the audience’s agreement with the judgment. If the audience finds the judgment *less warranted* for the prototype than for the actual case, the generalization will make the audience *agree less* with the judgment (negative prototype effect).

One thing should be added to this theory. If we look at cases where the judgment that the “prototype child” is less reliable as a witness is more warranted than the judgment that X is less reliable as witness, we quickly realize that they not only include cases where X is 12 years old, but also cases where X is 14, 17, 19, 25, and so on. In other words, they include cases where the audience will find it inappropriate to categorize X as a “child”. In situations where a category is used inappropriately there should be no (positive) prototype effect. On the contrary, there is reason to suspect that the audience will react negatively to the inappropriate use of the category and agree less with the judgment. In the following, we will refer to this kind of negative reaction as a *category transgression effect*.

To test if there is a positive and negative prototype effect as described above we conducted two experiments where the participants were presented with court room scenarios and asked to what extent they agreed with an argument that made use of the generalization. The first experiment tested the generalization “a child is less reliable as a witness” and the second experiment tested the generalization “a person who is intoxicated with alcohol is less reliable as a witness”. In each experiment the results were compared with a control group where the participants were presented with the same scenario and the same argument without the generalization.

## Experiment 1—“Child”

### Participants

Data was collected online using Amazon Mechanical Turk (http://www.mturk.com/)*,* an online service that recruits participants to carry out online tasks, such as surveys or experimental tasks. Amazon Mechanical Turk has an increasing popularity among social scientists as a source of data (Paolacci et al. 2010). A benefit of Amazon Mechanical Turk over traditional data collection methods is that it becomes feasible to run studies with large numbers of participants. Another benefit is that, compared to experiments where the participants consist of university students, participants on Amazon Mechanical Turk are more demographically diverse (Buhrmester et al. 2011; Ipeirotis 2010). The participants received $0.45 to complete the test. All participants were US residents. In total, 1187 participants took the test, but 45 participants were removed from the data set because they completed the test either in a very short time (less than 100 s was used as the exclusion criteria) or left the test incomplete. Left for the analysis were 1142 participants (577 women) with a mean age of 32 years (SD = 11.8).

### Design

A 6 × 2 fully factorial design was used. The first factor was the age of the witness in the court room scenario (4, 9, 12, 14, 17 or 19 years old). The second factor was the version of the argument made by the defense attorney (child generalization version and control version).

### Material

The experiment was based on a court room scenario with six versions. The six versions were identical, except for the age of the witness, Jessica Miller. Each participant was presented with one of the six versions. The court room scenario read as follows:

“Jason Williams is standing trial for murder. According to the prosecutor, Williams stabbed a man to death in front of a movie theatre in Atlanta, Georgia, on July 10, 2011. At the trial, the prosecution calls Jessica Miller as a witness. She is X and lives across the street from the movie theatre. She testifies that she saw the murder from her house, and identifies Williams as the killer.”

1. X = “4 years old”
2. X = “9 years old”
3. X = “12 years old”
4. X = “14 years old”
5. X = “17 years old”
6. X = “19 years old”

Then, the participants were presented with an argument from the defense attorney. Each scenario had two versions of the defense attorney’s argument, one version (child generalization) where the argument is based on the generalization “a child is less reliable as witness”, and one version (control) that does not use this generalization. In the control version, the word “child” is removed, and substituted with the age of Jessica Miller according to the scenario. Each participant was either given the child generalization version or the control version of the argument.

child generalization “I would like to draw your attention to one important circumstance regarding Jessica Miller. A child is less reliable as a witness. Jessica Miller is therefore less reliable as a witness.”
control “I would like to draw your attention to one important circumstance regarding Jessica Miller. A X is less reliable as a witness. Jessica Miller is therefore less reliable as a witness.”

1. X = “4-year-old”
2. X = “9-year-old”
3. X = “12-year-old”
4. X = “14-year-old”
5. X = “17-year-old”
6. X = “19-year-old”

### Procedure

After reading the given scenario and the given version of the argument the participants were asked to indicate their level of agreement with the argument on a scale from one (‘strongly disagree’) to nine (‘strongly agree’).

### Hypotheses

The purpose of the experiment was to investigate the difference in agreement with the argument in the child generalization version and the control version, over the six scenarios with the different ages of Jessica Miller. On the basis of the general theory outlined in Section 2 above our hypotheses for the experiment were as follows.

The participants will find the use of the category “child” appropriate if it is applied to a person whose age lies within the boundaries of their conception of “child”, and the participants will find the use inappropriate if it is applied to a person who is too old to be categorized as a child. This means that a division can be made between appropriate uses, where the age of Jessica Miller is below the *boundary age*, and inappropriate uses where her age is above the boundary age. We predicted that the participants would react negatively to inappropriate uses, and we therefore predicted that the participants would agree less with the argument in the child generalization version than the control version above the boundary age (category transgression effect).

For cases that fall below the boundary age a division can be made between cases where Jessica Miller is older than the prototype child and cases where she is younger than the prototype child. In cases where the age of Jessica Miller is higher than the prototypical age we expected the participants to agree more with the child generalization version than the control version (positive prototype effect). In cases where the age of Jessica Miller is lower than the prototypical age we expected the participants to agree less with the child generalization version than the control version (negative prototype effect).

**Fig. 1:**
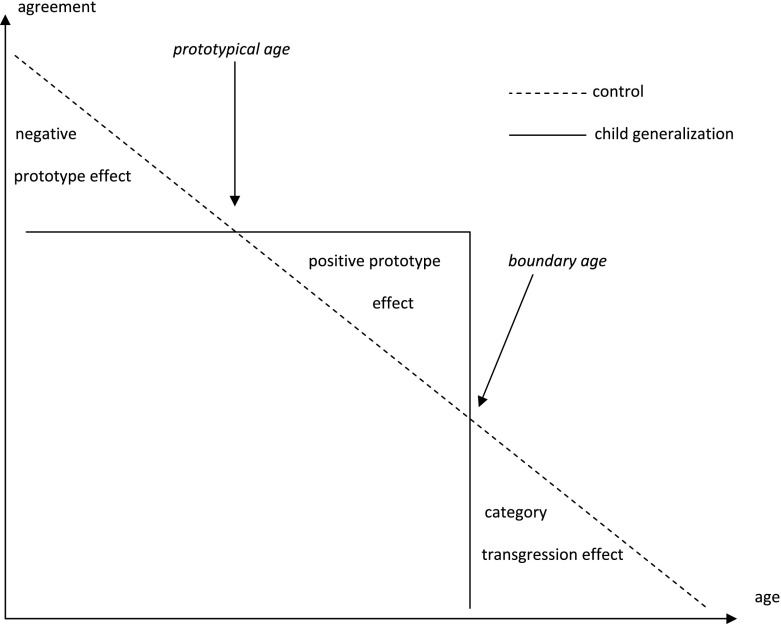
The mean agreement with the argument as a function of the age of the witness and argument version (hypotheses)

These hypotheses are conceptually displayed in Fig. 1, where the participant’s agreement with the argument is plotted against the age of Jessica Miller. In the control version, where the argument simply refers to Jessica Miller’s age, agreement declines monotonically with age (dotted line). In the child generalization version (straight line), agreement moves towards the degree of agreement at the prototypical age. And when the age of Jessica Miller passes the boundary age, agreement with child generalization version plummets sharply. It should be noted that the figure is idealized, and, in the real data, we would expect the boundary and the prototype to be fuzzy.

We assumed the prototypical age of a child to be around 7 years, and the boundary age to be around 15 years. On the basis of these assumptions we made the following predictions for the six scenarios. In Scenario 1 (4 years old) we predicted that the participants would agree less with the argument in the child generalization version than the control version (negative prototype effect). In Scenario 2 (9 years old), Scenario 3 (12 years old) and Scenario 4 (14 years old) we predicted that the participants would agree more with the argument in the child generalization version than the control version (positive prototype effect). In Scenario 5 (17 years old) and Scenario 6 (19 years old) we predicted that the participants would agree less with argument in the child generalization version than the control version (category transgression effect).

### Results

The results of the experiment are summarized in Table 1. To analyze the difference in agreement between the child generalization version of the argument and the control version, a *t*-test was conducted for each age.

**Table 1:** Means (and SDs), *t*-values, and corresponding Cohen’s *d* for agreement with the child generalization version and control version of the argument, for different ages of the witness

Age of witness	Child generalization		Control		*t*	Cohen’s *d*
	*N*	Mean and SD	*N*	Mean and SD		
4 years	89	5.31 (2.51)	95	5.84 (2.34)	−1.47	−.22
9 years	80	4.38 (2.21)	93	3.99 (2.22)	1.14	.18
12 years	89	3.98 (2.38)	96	3.13 (1.95)	2.67**	.39
14 years	95	2.92 (1.95)	99	2.37 (1.57)	2.13*	.31
17 years	94	2.21 (1.71)	99	2.16 (1.60)	.22	.03
19 years	108	1.59 (1.11)	105	2.12 (1.99)	−2.41*	−.25

*p < .05, **p < .01

In Scenario 1 (4 years old) participants agreed less with the argument in the child generalization version than the control version, as predicted (negative prototype effect). In Scenario 2 (9 years old), Scenario 3 (12 years old) and Scenario 4 (14 years old) participants agreed more with the child generalization version than the control version, as predicted (positive prototype effect). In Scenario 5 (17 years old) participants agreed equally with the argument in the child generalization version and the control version. This was not predicted. In Scenario 6 (19 years old) participants agreed less with the argument in the child generalization version than the control version, as predicted. As Table 1 shows the results were statistically significant in Scenario 3 (12 years old), Scenario 4 (14 years old) and Scenario 6 (19 years old).

**Fig. 2:**
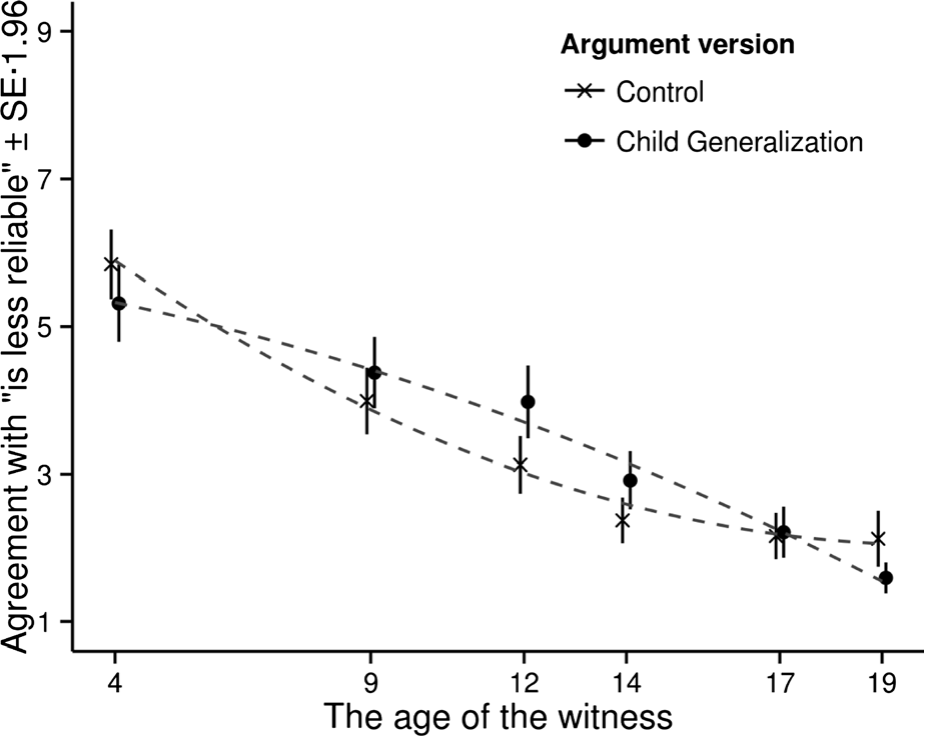
The mean agreement with the argument as a function of the age of the witness and argument version. The *dashed grey lines* show the best fitting quadratic curves

A graphic representation of the results is presented in Fig. 2. As we can see, Fig. 2 resembles the hypotheses depicted in Fig. 1.

The relation between the agreement rating and the age of the witness seems to be well captured by a quadratic function where the prototype effect is manifested as a difference in curvature between the curves for the two argument versions. A quadratic function that includes an interaction between the curvature of the relationship between the agreement rating and the age of the witness is also the most parsimonious model able to capture the hypothesized relationship shown in Fig. 1.

Linear regression modeling was used to test if there was a difference in curvature and, subsequently, a region with a positive prototype effect. Three models of increasing complexity were fitted to the data with the agreement rating as the response variable: A first model (M0) that included only an intercept term. A second model (M1) that also included the age of the witness as an explanatory variable (*x*_age_). A third model (M2) that included both the age of the witness and the argument version (*x*_arg_) as explanatory variables and that accounted for the difference in curvature between the two versions of the argument. These three models are summarized below:

$$ \begin{array}{l}\mathrm{M}0:{\upbeta}_0 + \upvarepsilon \hfill \\ {}\mathrm{M}1:{\upbeta}_0 + {\upbeta}_1{x}_{\mathrm{age}}+\upvarepsilon \hfill \\ {}\mathrm{M}2:{\upbeta}_0+{\upbeta}_1{x}_{\mathrm{age}}+{\upbeta}_2{x^2}_{\mathrm{age}}+{\upbeta}_3{x}_{\arg }+{\upbeta}_4{x}_{\mathrm{age}}{x}_{\arg }+{\upbeta}_5{x^2}_{\mathrm{age}}{x}_{\arg }+\upvarepsilon \hfill \\ {}\upvarepsilon \sim \mathrm{Normal}\left(0,\upsigma \right)\hfill \end{array} $$

The age of the witness variable was mean-centered before fitting the models to avoid multicollinearity due to the squared term. Comparing these three models using F-tests shows that M2 explains significantly more variance than M0 and M1 (*p* < 0.001). See Table 2 for details regarding this model comparison. All coefficients of M2 were significantly different from zero (see Table 3) which supports the difference in curvature visible in Fig. 1. The models with complexity between M1 and M2 were also compared with M1 using F-tests but none of these tests were significant.

**Table 2:** Comparisons between the three models

Model	Adjusted *R* ^2^	AICc	F-test model comparison	
			*F*	*P*
M0	–	3243.9	–	–
M1	.288	2857.8	469.0	<.001
M2	.299	2843.0	5.73	<.001

The F-tests shown on each row are between the model of the corresponding row and the model corresponding to the preceding row

**Table 3:** The fitted coefficients of model M2

	β_0_	β_1_	β_2_	β_3_	β_4_	β_5_
Estimate	2.8	−1.1	0.37	0.66	−0.26	−0.55
Std. error	0.12	0.091	0.088	0.17	0.13	0.13
*P*	<.001	<.001	<.001	<.001	<.05	<.001

**Fig. 3:**
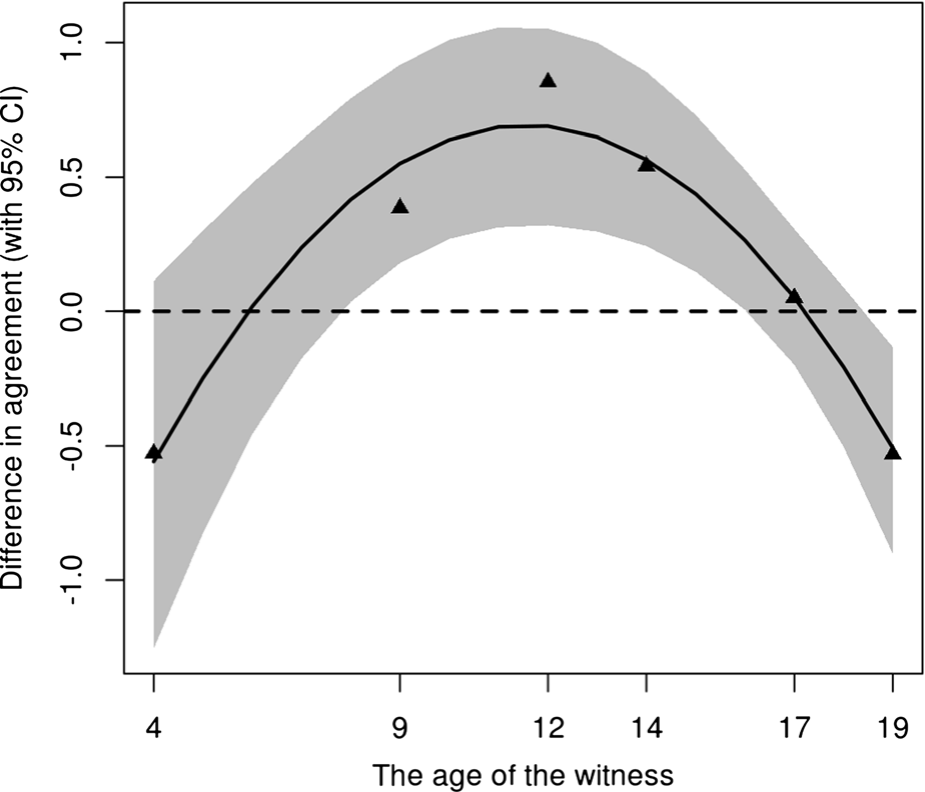
The mean difference in agreement between the two arguments version estimated by the model M2. A positive difference implies a positive prototype effect. The *grey region* shows the 95 % confidence region

Using M2, the strength of the prototype effect was calculated as the difference between the two curves shown in Fig. 2. This difference is shown in Fig. 3, together with a 95 % bootstrap confidence region (Efron 1979), where a positive difference implies a positive prototype effect. Taking the 95 % confidence interval as a measure of accuracy, there is a significant positive prototype effect in the 8–16 age range.

### Discussion

The results of the experiment support the idea that an argument that makes use of a generalization activates the prototype for the category used in the generalization, to the effect that the features of the prototype overshadow and partly overwrite the actual facts of the case. As we have seen, the case will to some extent be judged as if it had the features of the prototype instead of the features it actually has. This will enhance the audience’s agreement with the argument in situations where the audience finds the judgment more warranted for the prototype than for the actual case (positive prototype effect). But the use of generalizations can backfire. In situations where the audience finds the judgment more warranted for the actual case than for the prototype the activation of the prototype will decrease agreement (negative prototype effect). In such cases, the argument is more persuasive if the speaker refrains from the generalization and allows the facts to “speak for themselves”.

As we can see in Fig. 2, the child generalization curve and the control curve cross each other at approximately 7 years. If our general theory is correct, this suggests that the prototype child is about 7 years old, as we assumed when we formulated our predictions for Scenario 1 and 2. According to our theory, summarized in the hypothesis in Section 2, the audience will agree equally with both versions of the argument when the judgment for the prototype and the actual case are equally warranted.

The child generalization curve and the control curve also cross each other at approximately 17 years. If our general theory is correct, this suggests that the boundary age for “child” lies somewhere around 17 years, a little higher than we assumed when we formulated our predictions for Scenario 4, 5 and 6. The use of a generalization will only result in a positive prototype effect as long as the generalization is applied to a case that belongs to the category. If it is applied to case that falls outside the category the audience will react negatively to the inappropriate use of the generalization, as we saw in Scenario 6 (19 years old).

## Experiment 2—“Intoxicated”

### Participants

Data was collected from the same participants as the first experiment (see Section 3.1), using Amazon Mechanical Turk. The order of the questions asked in experiment one and experiment two was randomized for each participant.

### Design

A 6 × 2 fully factorial design was used. The first factor was the amount of wine consumed by the witness before he observed a murder (1, 2, 3, 4, 6 or 8 glasses). The second factor was the version of the argument made by the defense attorney (*intoxication generalization version* and *control version*).

### Material

The experiment was based on a court room scenario with six versions. The six versions were identical, except for the amount of wine that the witness, Leon Kowalski had consumed when he made his observations. Each participant was presented with one of the six versions. The court room scenario read as follows:

“Hector Hernandez is standing trial for the murder of Jamal Watts. According to the prosecutor, Watts was sitting on the front porch of his house at 3.30 in the afternoon of August 10, 2011 when Hernandez arrived in his car, slowed down and fired several shots at Watts through the open window. At the trial, the prosecution calls Leon Kowalski as a witness. Kowalski saw the murder from across the street. He testifies that he was walking down the street when he noticed a black sedan coming in his direction. The car slowed down in front of a house and he saw that the driver lifted a handgun and fired several shots at a man sitting in front of the house. Then the car took off. Kowalski identifies Hector Hernandez as the killer. When Kowalski was interrogated by the police right after the murder a police officer noted that Kowalski’s breath smelled of alcohol. Kowalski admitted that he had been drinking some wine at a local bar just before the shooting. Kowalski’s credit card records show that he drank X of red wine.”

1. X = “one glass”
2. X = “two glasses”
3. X = “three glasses”
4. X = “four glasses”
5. X = “six glasses”
6. X = “eight glasses”

Then, the participants were presented with an argument from the defense attorney. Each scenario had two versions of the defense attorney’s argument, one version (intoxication generalization) where the argument is based on the generalization “a person who is intoxicated with alcohol is less reliable as a witness”, and one version (control) that does not use this generalization. In the control version, the phrase “intoxicated with alcohol” is removed, and substituted with exact quantity of wine stated in the scenario. Each participant was either given the intoxication generalization version or the control version of the argument.

intoxication generalization “I would like to draw your attention to one important circumstance with regard to Leon Kowalski’s testimony. A person who is intoxicated with alcohol is less reliable as a witness. Leon Kowalski is therefore less reliable as a witness.”
control “I would like to draw your attention to one important circumstance with regard to Leon Kowalski’s testimony. A person who has consumed X of wine is less reliable as a witness. Leon Kowalski is therefore less reliable as a witness.”

1. X = “one glass”
2. X = “two glasses”
3. X = “three glasses”
4. X = “four glasses”
5. X = “six glasses”
6. X = “eight glasses”

### Procedure

After reading the given scenario and the given version of the argument the participants were asked to indicate their level of agreement with the argument on a scale from one (‘strongly disagree’) to nine (‘strongly agree’).

### Hypotheses

The purpose of the experiment was to investigate the difference in agreement between the intoxication argument and the control argument, over the six scenarios with the different quantities of wine consumed by the witness. On the basis of the general theory outlined in Section 2 above our hypotheses for the experiment were as follows. The participants will find the use of the category “intoxicated” appropriate if the quantity of wine consumed by the witness is high enough, and will find it inappropriate to categorize the witness as “intoxicated” if the consumed quantity is below the *boundary quantity* for intoxication. We predicted that the participants would react negatively to inappropriate uses, and we therefore predicted that the participants would agree less with the intoxication argument than the control argument below the boundary quantity (category transgression effect). For cases that fall above the boundary quantity a division can be made between cases where the quantity is lower than the quantity consumed by a prototypical intoxicated person and cases where the quantity is higher than the prototypical quantity. In cases where the quantity is lower than the prototypical quantity we expected the participants to agree more with the intoxication argument than the control argument (positive prototype effect). In cases where the quantity is higher than the prototypical quantity we expected the participants to agree less with the intoxication argument than the control argument (negative prototype effect).

**Fig. 4:**
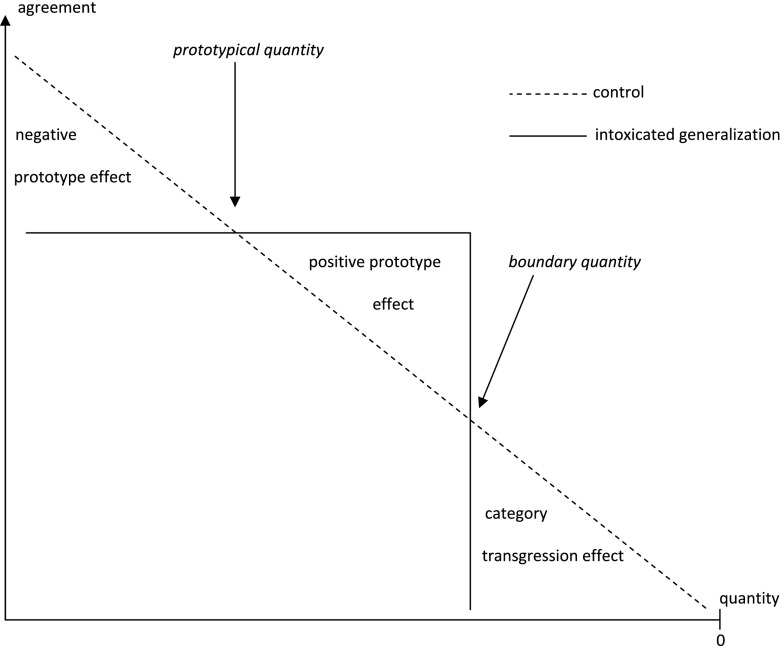
The mean agreement with the argument as a function of the quantity of wine and argument version (hypotheses)

These hypotheses are conceptually displayed in Fig. 4, where the participant’s agreement with the argument is plotted against the quantity of wine consumed by the witness. It should be noted that the quantity of wine decreases as we move to the right. The plotted line shows how a decrease in the consumed quantity of wine makes the argument against the witness less warranted.

We assumed the boundary quantity to lie between one and two glasses and the quantity consumed by a prototypical intoxicated person to be about five glasses of wine. On the basis of these assumptions we made the following predictions for the six scenarios. In Scenario 1 (one glass) we predicted that the participants would agree less with the intoxication argument than the control argument (category transgression effect). In Scenario 2 (two glasses), Scenario 3 (three glasses) and Scenario 4 (four glasses) we predicted that the participants would agree more with the intoxication argument than the control argument (positive prototype effect). In Scenario 5 (six glasses) and Scenario 6 (eight glasses) we predicted that the participants would agree less with the intoxication argument than the control argument (negative prototype effect).

### Results

The results of the experiment are summarized in Table 4. To analyze the difference in agreement between the intoxication generalization argument and the control argument, a *t*-test was conducted for each age.

**Table 4:** Means (and SDs), *t*-values, and corresponding Cohen’s d for agreement with the intoxication generalization version and control version of the argument, for different quantities of wine

Quantity of wine	Intoxication generalization		Control		*t*	Cohen’s *d*
	*N*	Mean and SD	*N*	Mean and SD		
1 glass	111	3.70 (2.37)	89	2.81 (1.73)	−3.08**	.61
2 glasses	99	4.33 (2.08)	91	3.73 (2.18)	−1.97*	.40
3 glasses	81	5.37 (2.33)	100	5.19 (2.25)	−.53	.11
4 glasses	117	5.96 (2.11)	77	6.03 (2.16)	.22	−.05
6 glasses	105	6.38 (1.94)	92	6.26 (2.29)	−.39	.08
8 glasses	85	6.66 (2.11)	95	6.97 (1.61)	.27	−.23

*p < .05, **p < .01

In Scenario 1 (one glass) participants agreed more with the intoxication argument than the control argument. This was not predicted. In Scenario 2 (two glasses) and Scenario 3 (three glasses) the participants agreed more with the intoxication argument than the control argument, as predicted (positive prototype effect). In Scenario 4 (four glasses) the participants agreed slightly less with the intoxication argument. This was not predicted. In Scenario 5 (six glasses) the participants agreed slightly more with the intoxication argument. This was not predicted either. In and Scenario 6 (eight glasses) the participants agreed less with the intoxication argument, as predicted (negative prototype effect). As Table 4 shows the results were statistically significant in Scenario 1 (one glass) and Scenario 2 (two glasses).

**Fig. 5:**
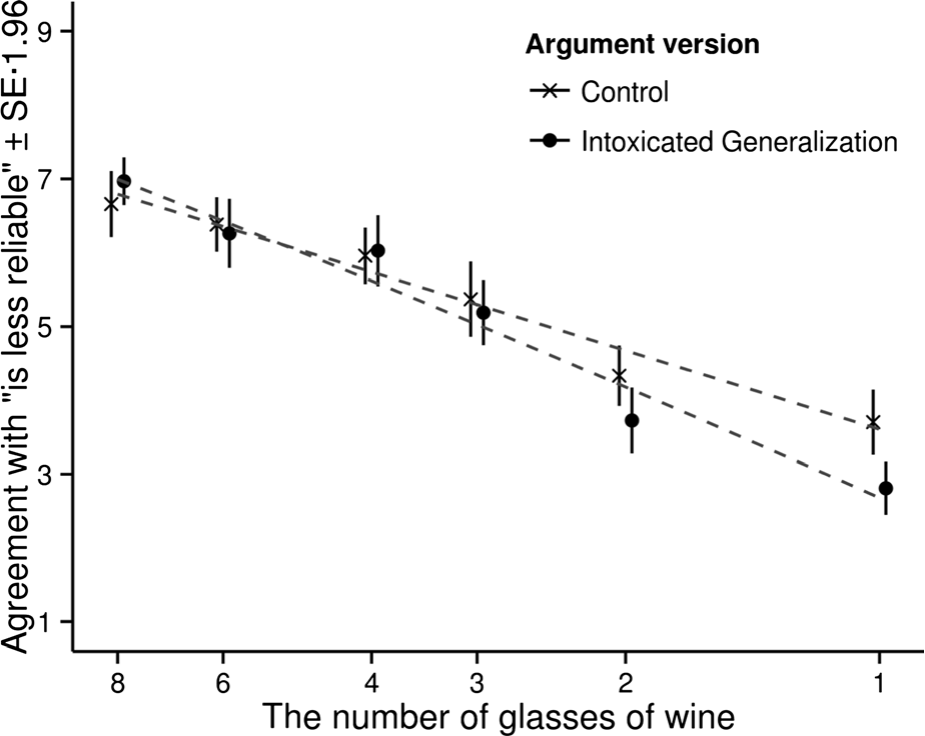
The mean agreement with the argument as a function of the quantity of wine and argument version. The *dashed grey lines* show the best fitting quadratic curves

A graphic representation of the results is presented in Fig. 5.

### Discussion

Some of the results matched our predictions, and some did not. There was a positive prototype effect in Scenario 2 and 3, as we predicted, but the graph (Fig. 5) did not match our hypotheses (Fig. 4) as good as the graph in Experiment 1 (Fig. 2) matched the hypotheses for that experiment (Fig. 1). At a low quantity of wine (1 glass), there was no category transgression effect, as we had predicted, and at a high quantity of wine (8 glasses), the negative prototype effect that we had predicted was small and not statistically significant. We had expected to see the curves cross each other twice (Fig. 4), one time at the boundary quantity and one time at the prototype quantity, but the results showed no boundary quantity cross over (right hand side of the graph), and the prototype cross over and negative prototype effect (left hand side) was small and not statistically significant.

An explanation for the lack of a boundary quantity cross over and a category transgression effect could be that the boundary quantity does not lie between one and two glasses, as we assumed, but is, actually, below one glass. This would explain why the curves do not cross on the right hand side. To create a situation where the participants find it inappropriate to categorize Leon Kowalski as intoxicated with alcohol, you need a scenario where Kowalski has consumed less than one glass of wine, for example a scenario where he has only consumed a teaspoon of wine. Since there was no such scenario in our experiment, we missed the boundary transgression effect.

That the predicted negative prototype effect on the left hand side was small and not statistically significant can be explained in a similar way. We assumed that the prototypical quantity consumed by an “intoxicated” is around five glasses, but it could be the case that it is, actually, around seven glasses. This would explain why we did not see a significant negative prototype effect in Scenario 6 (eight glasses). To see a significant effect, we should have tested a scenario with a higher quantity, e.g. a scenario where Kowalski has consumed ten glasses of wine.^1^

Notwithstanding this incompleteness in Experiment 2, there was a positive prototype effect in Scenarios 1, 2 and 3, and the effect was statistically significant in Scenario 1 (one glass) and Scenario 2 (two glasses). Experiment 2 therefore lends further support to our general theory that an argument that makes use of a generalization activates the prototype for the category used in the generalization, to the effect that the features of the prototype overshadow and partly overwrite the actual facts of the case.

## The Irrationality of the Prototype Effect

The prototype effects that we saw in the experiments can be taken as a sign of irrational behavior. Let us, for example, look at the positive prototype effect in Experiment 1, Scenario 3 (12 years old). The participants who were presented with the defense attorney’s argument in the child generalization version agreed significantly more than the participants who were presented with the argument in the control version. This difference in agreement is irrational, as the facts of the case were the same and the inference was the same. Both groups where given the same scenario, with the same information about the age of Jessica Miller, and both groups were presented with an argument from the defense attorney with the inference that Jessica Miller is less reliable as a witness because of her age. The only difference between the two versions of the argument was that the control version stated that “a 12-year-old is less reliable as a witness” while the child generalization version stated that “a child is less reliable as a witness”.

That a difference in agreement between the control version and the child generalization version is irrational becomes obvious if we imagine a person, X, who is presented with both versions, and disagrees with the control version but agrees with the child generalization version. The control version can be represented as a syllogism with “12-year-old” as the middle term:

- control version
  - [MAJOR PREMISE] A 12-year-old is less reliable as a witness.
  - [MINOR PREMISE] Jessica Miller is 12 years old.
  - [CONCLUSION] Therefore, Jessica Miller is less reliable as a witness.

The syllogism is logically valid according to *modus ponens*. To disagree with the argument advanced by the syllogism, X must claim that the major premise or the minor premise is incorrect. Since the correctness of the minor premise is given X must sustain that the major premise (“a 12-year-old is less reliable as a witness”) is incorrect. Bearing this in mind, let us now look at the child generalization version of the argument. The child generalization version is a syllogism where “child” serves as the middle term.

- child generalization version
  - [MAJOR PREMISE] A child is less reliable as a witness.
  - [MINOR PREMISE] Jessica Miller is a child.
  - [CONCLUSION] Therefore, Jessica Miller is less reliable as a witness.

To agree with this argument X must claim that the major premise as well as the minor premise is correct. Since X is committed to the view that it is incorrect that “a 12-year-old is less reliable as a witness” X is forced to interpret the major premise “a child is less reliable as witness” in a way that excludes 12-year-olds. X must interpret “child” to refer only to younger individuals, under the age of twelve. However, to maintain the correctness of the minor premise (“Jessica Miller is a child”) X is forced to interpret “child” in the minor premise to include 12-year-olds, as the given age of Jessica Miller is 12 years. This means that X assigns one meaning to the term “child” in the major premise and a different meaning to the term “child” in the minor premise. X commits a fallacy of rational thinking known as the *fallacy of equivocation* (Mill 1974; Quine 1950)*.* For a syllogism to be logically valid, the middle term must have the same meaning in both premises. Since X’s agreement with the child generalization argument is based on a fallacy of equivocation it is not an agreement in a rational sense. It is the kind of agreement that Arne Naess identified and analyzed as a *pseudo agreement* (Naess 1966).

A psychological explanation for why people fall for the *fallacy of equivocation* has been offered by John Woods and Douglas Walton. According to Woods and Walton, people have an innocent desire to interpret premises so that they become acceptable, and this desire overpowers the ability to notice that the argument slides from one meaning to another (Woods and Walton 2007).

## General Discussion

Our experiment shows what can happen when a case is put in a category. The category brings a certain prototype to mind, and the actual facts of the case are overshadowed by the prototype. This result is similar to observations that have been made in other studies. Elizabeth Loftus and John Palmer conducted an experiment where the participants watched films with car accidents, and where then asked about the incident they had seen (Loftus and Palmer 1974). Participants who were asked how fast the cars were going when they “smashed” into each other made a higher speed estimate than participants who were asked how fast the cars were going when they “bumped” into each other. Loftus and Palmer describe this memory effect as a “response bias” (Loftus and Palmer 1974, 586), but it could just as well be explained as a prototype effect. The question “How fast were the cars going when they smashed?” categorizes the case as a “smash” to the effect that the actual speed of the cars is overshadowed by the prototypical speed of a “smash”. The effect has been replicated in later studies (MacLin 2002).

There is an important difference between the effect that Loftus and Palmer uncovered in their experiment and the effect that we have observed in this study. The effect studied by Loftus and Palmer is a memory effect. Participants were manipulated in their memory of an event (the speed of a car). The effect observed in our study is not a memory effect, as the participants had information about the exact age of the witness (Experiment 1) and the amount of wine consumed (Experiment 2) right in front of them, explicitly stated above the question. Our study is concerned with evaluation, not memory. Participants were manipulated to agree with a certain evaluation (the witness is not reliable). These effects are useful for different purposes in legal rhetoric. The memory effect observed by Loftus and Palmer can be used by a skilled litigator in the questioning of a witness, to manipulate the memory of the witness. The prototype effect observed in our study can be used by the litigator in his arguments to the jury, to manipulate the jurors in their evaluation of the evidence. A litigator who seeks to prove that a car accident was caused by the defendant’s negligence can categorize the accident as a “smash” when he asks the witness how fast the defendant was going, to make the witness “remember” the speed as higher than it actually was. A litigator who seeks to undermine the reliability of a witness can categorize the witness as a “child”, to make the jurors think of her as younger than she actually is when they evaluate her reliability.

This kind of manipulation can lead to incorrect and unjust verdicts. Are such litigation strategies legal? With regard to the manipulation of witness memory there is a provision in the Federal Rules of Evidence that prohibits “leading questions” (FRE 611c). According to this rule, a litigator shall not formulate questions to the witness in a way that suggests a certain answer. This rule can be used by the opposing council to voice an objection against a litigator who uses a suggestive categorization (“How fast did the car go when it smashed into the other car?”) If the judge sustains the objection, the litigator will have to reformulate his question. In contrast to this, there is no procedural rule that prohibits a litigator from using suggestive categorizations to manipulate the jurors in their evaluation of the evidence. A litigator is free to use any categorization and generalization that suits his case, and many litigators do. A litigator is free to categorize a 12 year old as a “child”, a person who has consumed one glass of wine as “intoxicated”, a person who was caught shoplifting once in his youth as a “convicted felon”, a person who has worked for 6 months as a doctor as an “experienced physician”, and so on.

## Conclusions

An argument that makes use of a generalization activates the prototype for the category used in the generalization. We conducted two experiments that investigated how the activation of the prototype affects the persuasiveness of the argument. The results of the experiments suggest that the features of the prototype overshadow and partly overwrite the actual facts of the case. The case is, to some extent, judged as if it had the features of the prototype instead of the features it actually has. This prototype effect increases the persuasiveness of the argument in situations if the audience finds the judgment more warranted for the prototype than for the actual case (positive prototype effect), but decreases persuasiveness in situations where the audience finds the judgment less warranted for the prototype than for the actual case (negative prototype effect).

## Footnotes

1 The absence of a significant negative prototype effect could also be explained in a different way. It could be the case that Experiment 2 came out differently than Experiment 1 because “intoxicated” is a different kind of concept than “child”. It could be the case that a person always comes closer to being typically “intoxicated” for every glass that he drinks, no matter how many glasses he has consumed before. In other words, there is no quantity that exceeds the quantity that first comes to mind when we hear that someone is “intoxicated with alcohol”. If this is correct, the prototypical quantity lies at the extreme end of the scale, and there is no quantity where the generalization is less warranted for the prototype than the actual case. This would explain the absence of a significant negative prototype effect. With a category like this there can be no negative prototype effect. An objection against this explanation is that, if it were true, the first thing that would come to mind, when we hear that someone is “intoxicated with alcohol”, would be that the person is in a state of delirium, and this does not seem to be the case.
